# Instrumentation-Related Complications Following Nonfusion Posterior Fixation in Patients with Metastatic Spinal Tumors: Incidence and Risk Factors

**DOI:** 10.3390/jcm14134629

**Published:** 2025-06-30

**Authors:** Yunjin Nam, Jin-Sung Park, Dong-Ho Kang, Chong-Suh Lee, Seung Woo Suh, Se-Jun Park

**Affiliations:** 1 Department of Orthopedic Surgery, Korea University Guro Hospital, Seoul 08308, Republic of Korea; 2 Department of Orthopedic Surgery, Samsung Medical Center, Seoul 06351, Republic of Korea; 3 Department of Orthopedic Surgery, Haeundae Bumin Hospital, Busan 48094, Republic of Korea

**Keywords:** metastatic spinal tumor, posterior fixation, nonfusion, instrumentation related complications, risk factors, survival analysis

## Abstract

**Background/Objectives**: Previous studies have reported satisfactory outcomes and low rates of instrumentation-related complications (IRCs) following nonfusion posterior fixation in patients with metastatic spinal tumors (MSTs). However, to adequately assess the longevity and durability of nonfusion instrumentation in patients with longer life expectancy, an extended follow-up period is essential. This study aims to evaluate the incidence of and risk factors for IRCs in patients with MSTs who underwent nonfusion posterior fixation and had radiographic follow-up data available for at least one year postoperatively. **Methods**: Consecutive data were collected from patients who underwent pedicle screw-based posterior fixation without fusion for MSTs in the thoracic and/or lumbar region from 2005 to 2018. The IRCs included screw loosening, screw pull-out, and metal breakage. The IRC-free survival and related factors were analyzed by Kaplan–Meier survivorship analysis with the log-rank test within a minimum follow-up period of one year. A multivariate analysis was performed using a Cox proportional-hazards regression model. **Results**: In total, 61 patients were included. The mean follow-up period was 28.3 months (range: 12.0–102.6 months). There were 27 cases (44.2%) of IRCs, including 22 cases of screw loosening, four cases of screw pull-out, and one case of rod breakage, at an average of 9.6 months (range: 1.0–38.1 months). The median IRC-free survival was 38.1 months (range: 1.0–102.6 months). Only three patients experienced pain aggravation with IRCs. No revision surgery was performed. A multivariate analysis identified that fixation length was a risk factor for IRCs (odds ratio: 0.358, 95% confidence interval: 0.114–0.888; *p* = 0.027). **Conclusions**: IRCs are frequent but mostly asymptomatic after nonfusion posterior fixation in patients with MSTs followed up for at least one year. Overall, the IRC-free survival was long enough considering the patient survival. Fixation length was a significant risk factor for IRCs regardless of MST location.

## 1. Introduction

As the overall survival profile of cancer patients improves with continued advancements in medical treatment, the survival of patients with metastatic spinal tumors (MSTs) is also increasing [1]. Considering that the natural course of untreated MSTs would be unfavorable with time, those patients may experience pain or neurologic deficits related to MSTs [2,3]. Surgical treatment has been adopted more frequently than ever before to treat or prevent these morbidities in patients with MSTs [4]. Stabilization and/or decompression are the most popular surgical procedures included in palliative therapy plans [2].

Spinal fusion has been frequently paired with instrumentation to increase construct longevity and avoid late failures of instrumentation such as screw loosening, screw pull-out, or metal breakage [5,6]. In many patients with MSTs, however, the fusion procedure is difficult to complete due to a lack of adequate fusion bed or the individual’s poor general condition. Additionally, fusion is difficult to achieve because of the effects of perioperative radiotherapy, chemotherapy, steroid use, and malnutrition [7]. Patients with MSTs usually have a relatively short life expectancy, which might not be long enough to achieve solid bony fusion [4]. For these reasons, spinal instrumentation without fusion has been considered in the management of MSTs.

Recent studies have reported favorable outcomes and low rates of instrumentation-related complications (IRCs) following nonfusion instrumentation in patients with MSTs [7,8,9]. However, many of these studies were limited by relatively short follow-up durations, making it difficult to assess the long-term durability of nonfusion constructs. Additionally, radiographic confirmation of instrumentation status at final follow-up was not consistently described. Evidence regarding IRC-free survival and associated risk factors in patients undergoing nonfusion surgery remains limited, particularly in those with longer life expectancy.

The current study aims to investigate the incidence of and risk factors for IRCs after posterior fixation without fusion in patients with MSTs. To evaluate long-term construct durability, we included a relatively large cohort of patients whose radiographic follow-up was available for at least 12 months after surgery.

## 2. Materials and Methods

### 2.1. Study Design

This study was a single-center, retrospective study performed using medical records. A total of 278 patients underwent surgical treatment for MSTs between 2005 and 2018. The main reasons for surgery in this group were severe axial pain with or without radiating pain or neurologic deficit. Decisions about whether to pursue surgical treatment and regarding which surgical option were made following a full discussion amongst the spine tumor board composed of medical oncologists, radiation oncologists, and spine surgeons.

To ensure consistency in the surgical technique analyzed, we first limited the cohort to those with thoracic or lumbar MSTs treated using pedicle screw fixation. This anatomical restriction excluded 51 patients with cervical or sacral lesions. Among the remaining 227 patients, 75 patients survived for more than one year based on their date of death or last follow-up visit. Among these, only those with radiographic follow-up—either plain radiographs or computed tomography (CT) scans—available at 12 months postoperatively were included.

CT imaging was not routinely performed but was included if chest or abdominopelvic CT scans taken for oncologic purposes included the operated spinal levels. Based on this criterion, 14 patients without appropriate imaging at the 12-month mark were excluded, resulting in a final cohort of 61 patients.

### 2.2. Surgical Procedures

The surgeries were performed by five spine surgeons at a single institute. All surgeries were performed using pedicle screw-based posterior fixation without any fusion procedures. The type of surgical procedure was determined by the spine tumor board by considering several factors, such as the main symptom of patients, the primary tumor site, and the location and extent of MSTs. For this study, we classified the surgical procedures chosen into three categories: posterior fixation without decompression, posterior fixation with laminectomy, and posterior fixation with debulking. Although the surgical procedures were determined variously by the surgeons, only posterior fixation was usually performed when radiographic instability was observed and mechanical pain was the main symptom without prominent neural compromise. In such cases, both open and percutaneous approaches were used. The choice between them was primarily guided by tumor location and anatomical accessibility: open fixation was preferred for thoracic lesions, whereas percutaneous fixation was more common for thoracolumbar junction and lumbar levels. For those with acute or subacute neurologic deficits with evidence of tumor invasion to the spinal canal, posterior fixation with laminectomy or debulking procedures were conducted. Debulking was performed via an anterior or posterior approach in patients with more favorable primary tumors and who were thought to be able to tolerate the aggressive surgery. When completing vertebrectomy during debulking surgery, the vertebral body was replaced by bone cement alone or bone cement within a mesh cage.

### 2.3. Outcome Measures

**Figure 1:**
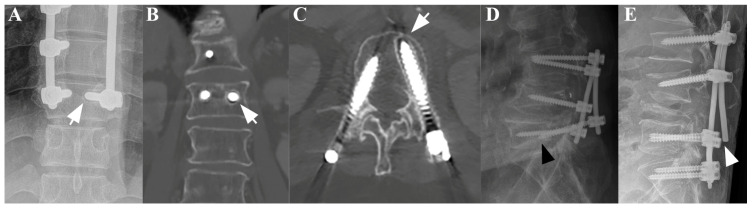
A plain anteroposterior (AP) radiograph (**A**) and coronal (**B**) and axial (**C**) CT scans of a patient subjected to posterior fixation showing radiological signs of screw loosening, indicated by the white arrows. Separately, plain lateral radiographs of a patient subjected to posterior fixation showing radiological signs of screw pull-out ((**D**), black arrowhead) and rod breakage ((**E**), white arrowhead).

The main outcomes in this study were IRCs, which included screw loosening, screw pull-out, and metal breakage (Figure 1). IRCs were determined based on follow-up imaging at or beyond 12 months postoperatively using either plain radiography or CT imaging. Routine radiographic surveillance was conducted at approximately 3, 6, and 12 months postoperatively, followed by annual imaging. As in previous research, screw loosening was defined as the presence of a radiolucent zone measuring more than 1 mm surrounding the screw on the plain radiograph or CT scan [10]. Screw pull-out was defined as a 5° or greater change in the angle formed by the screw direction and upper endplate. Metal breakage was defined as any discontinuities in the implants. All imaging assessments were performed independently by one spine surgeon and one radiologist. Discrepancies were resolved by consensus or, when unresolved, based on the radiologist’s interpretation.

When IRCs were observed during follow-up, an assessment of whether the pain was aggravated or not around the time of IRC identification was completed.

Risk factors for IRCs were evaluated according to the following variables: sex, age (<60 vs. ≥60 years), body mass index (<18.5, 18.5–24.9, or ≥25.0 kg/m^2^), primary tumor site, number of vertebrae with metastases (1, 2, or ≥3 vertebrae), Spinal Instability Neoplasm Score criteria (stable, potentially unstable, or unstable) [11], location of metastases (at or above T10 vs. below T10), history of radiotherapy, location of the lowest instrumented vertebra (LIV) (at or above T10 vs. below T10), preoperative and postoperative Eastern Cooperative Oncology Group (ECOG) scale (0–2 vs. 3–4), type of surgical procedure (posterior fixation only, posterior fixation with laminectomy, or posterior fixation with debulking procedure), fixation length [number of instrumented vertebrae without metastases (<3 vs. ≥3)] [12], screw density [number of pedicle screws per number of pedicles within the instrumented level (<0.67 or ≥0.67)] [13], and fixation method (open vs. percutaneous).

The presence or absence of metastases in each vertebra was primarily determined by magnetic resonance imaging (MRI), which served as the main diagnostic modality for evaluating tumor involvement.

### 2.4. Statistics

For univariate analysis, the presumed risk factors were compared in terms of IRC-free survival between patients with and without IRCs using Kaplan–Meier survivorship analysis with the log-rank test. Using the variables found to be significant with *p*-values of less than 0.05 during univariate analysis, a multivariate analysis was performed using a Cox proportional-hazards regression model. A forward stepwise procedure was adopted for the multivariate analysis. The statistical analysis was performed using the Statistical Package for the Social Sciences software program version 25.0.0 (IBM Corp., Armonk, NY, USA). A *p*-value of less than 0.05 was considered to be statistically significant.

## 3. Results

The study cohort consisted of 61 patients (37 men and 24 women, mean age of 61.7 years). The demographic and clinical data are described in Table 1.

There were no statistically significant differences in sex (*p* = 0.726) or age group (*p* = 0.266) between the included patients (n = 61) and those excluded due to insufficient follow-up, which was defined as either death within one year or absence of radiographic imaging at 12 months (n = 166). However, the distribution of primary tumor types showed a statistically significant difference (*p* < 0.001), suggesting that tumor biology may have influenced patient survival or follow-up availability. Additionally, no statistically significant differences were observed in sex (*p* = 1.000), age group (*p* = 0.250), or primary tumor type distribution (*p* = 0.634) between the included patients (n = 61) and those specifically excluded due to lack of imaging despite surviving longer than one year postoperatively (n = 14), indicating that radiographic loss to follow-up among long-term survivors was unlikely to have introduced meaningful selection bias.

The mean follow-up duration was 28.3 months (range: 12.0–102.6 months). At the point of final follow-up, 29 patients remained alive. The median overall survival after surgery was 35.6 months (95% CI, 21.2–50.0 months). Twenty-seven of these patients (44.1%) experienced IRCs at a mean of 9.6 months after surgery (range: 1.0–38.1 months). IRCs occurred less than one year after surgery in 19 cases and more than one year after surgery in eight cases. The most common IRC was screw loosening (n = 22 cases). The others included four cases of screw pull-out and one case of rod breakage. There were no cases characterized by screw breakage. Two cases of IRCs (one of screw loosening, one of screw pull-out) were accompanied by vertebral body fracture. Of these 27 patients with IRCs, only three patients (two with screw loosening, one with screw pull-out) experienced pain aggravation related to their respective IRCs. A patient with screw loosening accompanied by vertebral body fracture underwent cement augmentation. No revision surgeries were performed to address any of the IRCs.

The risk factors related to IRCs were analyzed using the log-rank test of the Kaplan–Meier survivorship analysis (Table 2).

**Figure 2:**
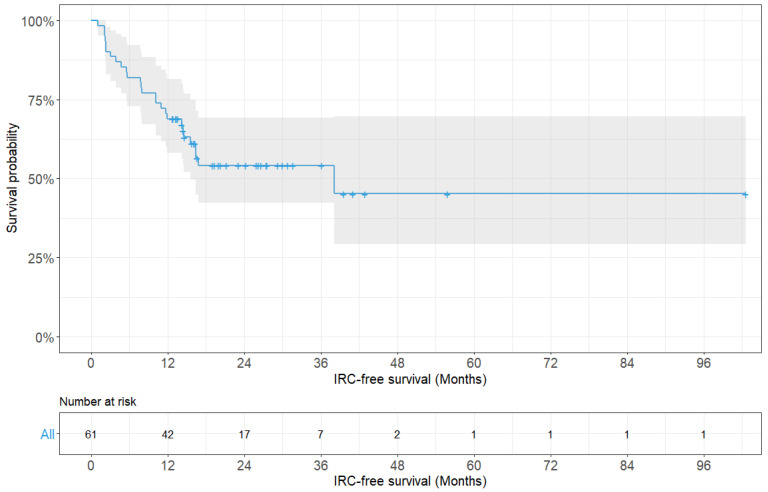
Kaplan–Meier survivorship curve showing instrumentation-related complication-free survival probability for all patients (n = 61).

History of radiotherapy, fixation length, and screw density were significantly associated with IRCs (*p* = 0.042, *p* = 0.017, and *p* = 0.027, respectively). Other variables, such as sex, age, primary tumor type, ECOG performance status, BMI, and fixation method showed no significant association with IRCs. A multivariate analysis using Cox proportional-hazards regression was carried out, involving the variables found to be statistically significant during the univariate analysis, and it revealed that only the fixation length was a significant risk factor for IRCs (hazard ratio: 0.358, 95% CI: 0.144–0.888; *p* = 0.027). The median IRC-free survival was 38.1 months (range: 1.0–102.6 months) (Figure 2).

**Figure 3:**
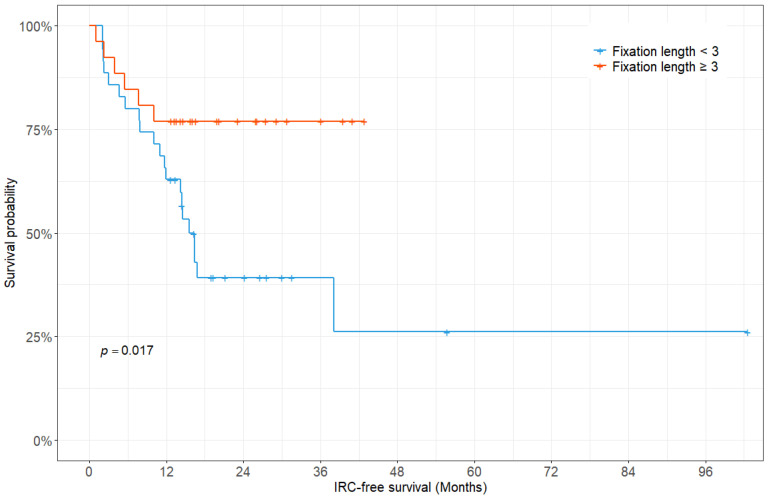
Kaplan–Meier survivorship curve showing instrumentation-related complication-free survival fraction according to the fixation length.

There was a significant difference in the median IRC-free survival according to the fixation length (15.6 months for a fixation length of less than three vertebrae vs. not reached for a fixation length of three or more vertebrae; *p* = 0.017, log-rank test) (Figure 3).

There was a significant difference in the incidence of IRCs according to the fixation length when the location of the LIV was at or above T10 (8.3% for fixation length < 3 vs. 63.6% for fixation length ≥ 3, *p* = 0.007) (Table 3).

### 3.1. Illustriative Cases

#### 3.1.1. Case 1

**Figure 4:**
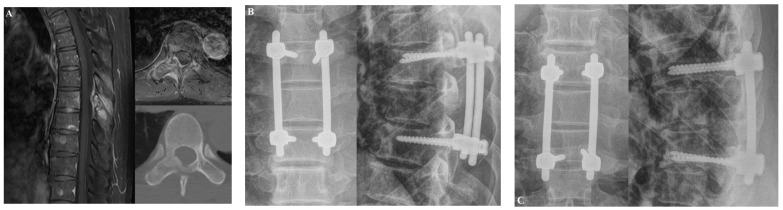
Illustrative case 1. A 58-year-old man was diagnosed with non-small-cell lung cancer metastasis to T7. The patient suffered from mechanical back pain. (**A**) Preoperative T1-weighted enhanced magnetic resonance imaging (MRI) and axial CT scans revealed T7 metastasis. (**B**) At three months after laminectomy with posterior fixation at T6 to T8, his back pain had improved. (**C**) Screw loosening occurred without pain aggravation approximately 12 months after the operation.

The first patient was a 58-year-old man who was diagnosed with T7 metastasis associated with non–small-cell lung cancer (Figure 4). The T7 metastasis resulted in mechanical back pain. The patient underwent T7 laminectomy and fixation from T6 to T8. Postoperatively, his back pain improved. However, at approximately 12 months after surgery, screw loosening occurred without pain aggravation. At the last follow-up visit (5.8 years after surgery), the patient reported experiencing no significant back pain.

#### 3.1.2. Case 2

**Figure 5:**
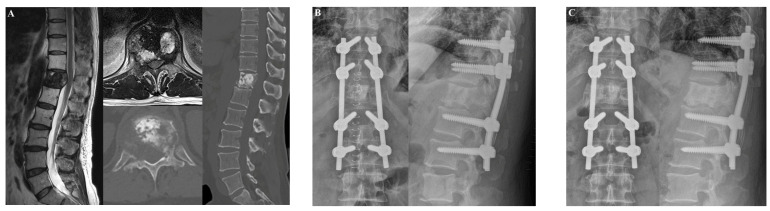
Illustrative case 2. A 60-year-old man was diagnosed with non–small-cell lung cancer metastasis to T12. The patient suffered from buttock pain. (**A**) Preoperative T2-weighted MRI and CT scans revealed T12 metastasis. (**B**) At two weeks after laminectomy with posterior fixation from T10 to L2, the buttock pain improved. (**C**) At the last follow-up, 18 months after the operation, there was no evidence of instrumentation-related complications.

The second patient was a 60-year-old man who was diagnosed with T12 metastasis associated with non–small-cell lung cancer (Figure 5). The patient had a history of palliative radiotherapy for thoracolumbar spinal metastases (two months prior). The spinal metastasis resulted in right buttock pain that did not subside after the palliative radiotherapy. After laminectomy and fixation from T10 to L2, the buttock pain improved and the patient could walk for an hour. There was no evidence of IRCs at the last follow-up visit (1.5 years after surgery).

## 4. Discussion

Obtaining solid bony fusion following instrumentation is considered essential for nononcologic patients to achieve increased construct longevity and better outcomes [5,14]. When spinal fusion is not performed after spinal instrumentation, the risk of developing IRCs increases [15,16]. IRCs, including screw loosening, screw pull-out, and metal breakage, are associated with fatigue on the instrumentation [17]. After spinal fixation, the micromotion within fixed segments exerts repetitive loading on instrumentation [18]. If the fusion procedure is not performed or if solid fusion is not obtained after the fusion procedure, the fatigue applied to the instrumentation accumulates over time and the risk of IRCs increases. Mohi et al. reported that most cases of instrumentation failure occurred before obtaining solid bony fusion [17]. However, fusion procedures can be difficult to perform, and solid bony fusion can be difficult to obtain after the fusion procedure in many patients with MSTs [7]. A longer operation time is necessary, with more bleeding associated with the decortication procedure, and greater costs are associated with the fusion materials [18,19]. Therefore, there is controversy as to whether fusion should be performed in patients with MSTs [8]. Recent studies have reported that satisfactory results after fixation-only surgery can be achieved [7,8,9,20].

The survival of patients with MSTs varies according to several factors including primary tumor, performance status, and the number of visceral metastases [21]. Chang et al. observed that patients with MSTs survived more than three years after spinal metastases were diagnosed [22]. Shehadi et al. reported that the median survival after the first surgery in patients with MSTs from breast cancer, who are known to have good prognoses, was 21 months (95% CI: 16–27 months) [23]. As systemic treatment has developed, the survival of patients with MSTs has lengthened [1,24], and further studies are required to evaluate whether nonfusion fixation surgery can provoke longevity in patients with MSTs. To evaluate the usefulness of nonfusion surgery as an alternative to fusion surgery, a follow-up period of at least one year, which is longer than the period necessary to achieve solid fusion [25], is required.

In the current study, IRCs occurred in 27 of 61 patients (44.1%) during the mean follow-up period of 28.3 months. Among these, screw loosening was the most common (n = 22 cases). Previous studies have reported that patients with spinal metastases tend to have higher rates of IRCs compared to those with degenerative spinal disorders. This increased risk is believed to be attributable to poor bone quality, systemic disease burden, and the effects of adjuvant therapies such as radiotherapy and chemotherapy. While the reported incidence of IRCs in degenerative spine disease ranges from 0.85% to 10.4% [26,27], studies in metastatic spine tumor patients have shown a higher incidence, typically ranging from 10% to 40% [8,28]. Our findings are consistent with this trend, demonstrating a relatively high frequency of IRCs in this patient population. When comparing the incidence of IRCs between the early (2005–2012) and late (2013–2018) periods, no significant difference was observed (46.2% vs. 43.8%). In a previous study, we reported nine cases (6.4%) of IRCs in 140 operations and three cases (2.2%) of symptomatic IRCs in 136 patients during a mean follow-up period of 16.5 months [9]. Drakhshandeh et al. reported zero cases of IRCs in 27 patients during a mean follow-up period of 12.2 months [8]. There were reasons for why the incidence of IRCs in the present study was much higher than that in previous studies. First, our present study had a longer mean follow-up period relative to those of other studies. Second, the definition of IRCs varied. Drakhshandeh et al.’s study did not include screw loosening in the definition of IRCs. In the current study, the number IRCs excluding screw loosening totaled five cases (8.1%), which is not significantly different from those reported in previous studies. Of the 61 patients in the current study, three (4.9%) experienced pain related to IRCs at a mean of 4.3 months after surgery, although only one patient (1.6%) underwent intervention via cement augmentation. Despite the majority of the IRCs being asymptomatic, initial instrumentation was considered necessary to achieve spinal stability and prevent neurologic deterioration in this vulnerable patient population. Amankulor et al. reported nine cases of symptomatic IRCs in 318 patients and a median of 39.9 months of IRC-free survival [28]. Even though the follow-up period of our study was longer than that of other studies, it was not significantly different when compared to previous studies. The median IRC-free survival period was 38.1 months, whereas the median overall survival period was 35.6 months. Even the median overall survival period after surgery in our study was longer than in previous studies [22,23], while the median IRC-free survival length exceeded the median overall survival length. This suggests the longevity of fixation surgery without fusion in patients with MST.

In our study, the primary risk factor for IRCs was fixation length, which was defined as the number of instrumented vertebrae without metastases, because the number of vertebrae with metastases also affected the construct length. When one-above and one-below pedicle screw fixation over vertebrae with metastases was performed, the fixation length was two. A fixation length of three or more vertebrae significantly decreased the risk of IRCs. The long fixation length increased the stability in the spinal column, while the short fixation length increased the construct failure and loss of correction [29]. Amankulor et al. reported that construct length was one of the risk factors for IRC onset after surgery in patients with MSTs [28].

Although adopting a long fixation length reduces the risk of IRCs, it has several disadvantages. For example, it loses more motion segments, affects adjacent segments with larger bending moments, and necessitates more operation time to complete. Since the thoracic spine has more stiffness than the lumbar spine because of the presence of the rib cage [30], the adequate fixation length will vary depending on the location of the construct. In the current study, if the fixation length was three or more vertebrae, the incidence of IRCs was decreased relative to when the fixation length was less than three vertebrae, regardless of the location of the LIV. However, when the fixation length was three or more vertebrae, the incidence of IRCs was significantly smaller when the LIV was located at or above T10. The incidence of IRCs might be decreased in the thoracic region because the thoracic spine achieves less bending movements due to the presence of the rib cage. McLain et al. reported that the involvement of a long fixation construct has an advantage in the thoracic area, whereas shorter fixation constructs have an advantage in the lumbar area [31].

Several limitations should be acknowledged. The small sample size and retrospective design limit the statistical power of the study and introduce a potential risk of information bias. Additionally, this study may have overestimated the overall survival and IRC-free survival, as patients with less than one year of follow-up were excluded. This exclusion was intentional, as our primary aim was to assess the long-term mechanical durability of nonfusion constructs. Early postoperative complications may be affected by systemic factors such as disease progression or surgical complexity rather than construct mechanics. Including patients who died early may have introduced confounding factors unrelated to implant performance. The radiographic evaluations were not standardized, with both plain radiographs and CT scans used. Notably, the IRCs may have been underestimated because CT imaging is less sensitive than plain radiography for detecting screw loosening [32]. Although bone mineral density (BMD) was not routinely measured in the included patients and therefore not analyzed, it may influence the risk of instrumentation-related complications. We acknowledge the absence of BMD data as a limitation. Likewise, systemic treatments such as bisphosphonates, immunotherapy, or targeted therapy were not specifically considered in the analysis. Although these therapies can potentially affect bone healing and implant stability, detailed treatment data were not uniformly available in the medical records. We acknowledge this as another limitation of our study. Furthermore, cement augmentation, or kyphoplasty, was not performed in any of the included cases. Considering the relatively high incidence of IRCs such as screw loosening, these adjunctive procedures may have provided additional construct stability. Their potential role should be explored in future studies, particularly in patients with compromised bone quality. Likewise, spinal alignment was not independently analyzed as a variable; however, it may have been indirectly accounted for through the SINS criteria, which include components related to deformity. Implant size, particularly screw diameter, was not statistically assessed, although the largest possible pedicle screws were used intraoperatively based on surgeon judgment. Furthermore, this study focused solely on radiographic outcomes and did not include data on clinical outcomes. Finally, because patients who underwent fusion with instrumentation were not included, a direct comparison between nonfusion and fusion surgery was not possible.

Given that the mean IRC-free survival was 9.6 months and that most of the IRCs occurred within the first postoperative year, we recommend routine radiographic surveillance at 3, 6, and 12 months postoperatively, followed by annual imaging. If IRC is identified at 6 months or if symptoms are present, interim imaging at 9 months may be beneficial to monitor progression or symptom correlation.

## 5. Conclusions

The current study reported that IRCs were frequent but mostly asymptomatic following posterior fixation without fusion in patients with MSTs followed up for at least one year. The IRC-free survival was long enough considering the actual survival of patient. Longer fixation lengths of three or more vertebrae reduced IRCs regardless of the MST location, but the IRCs were more significantly reduced when the location of the LIV was at or above T10.

## Figures and Tables

**Table 1:** Demographic data of patients.

Variable	Value
Sex	
Male	37 (60.7%)
Female	24 (39.3%)
Age; mean ± SD, range (years)	61.7 ± 10.5, 31–83
<60	22 (36.1%)
≥60	39 (63.9%)
Body mass index (kg/m^2^)	
<18.5	1 (0%)
18.5–24.9	38 (62.3%)
≥25	22 (37.7%)
Primary tumor site	
Lung	14 (23.0%)
Kidney	12 (19.7%)
Breast	8 (13.1%)
Liver	6 (9.8%)
Prostate	5 (8.2%)
Thyroid	4 (6.6%)
Colorectal	2 (3.3%)
Miscellaneous	10 (16.4%)
Number of involved vertebral bodies	
1	49 (80.3%)
2	7 (11.5%)
≥3	5 (8.2%)
SINS criteria *	
Stable	2 (3.3%)
Potential unstable	52 (85.2%)
Unstable	7 (11.5%)
Location of metastases	
At or above T10	27 (44.3%)
Below T10	34 (55.7%)
History of radiotherapy	
Yes	55 (90.2%)
No	6 (9.8%)
Preoperative ECOG scale	
0–2	40 (65.6%)
3–4	21 (34.4%)
Postoperative ECOG scale	
0–2	54 (88.5%)
3–4	7 (11.5%)
Location of the LIV	
At or above T10	23 (37.7%)
Below T10	38 (62.3%)
Type of surgical procedure	
Posterior fixation only	19 (31.1%)
Posterior fixation with laminectomy	25 (41.0%)
Posterior fixation with debulking procedure	17 (27.9%)
Fixation length †	
<3	35 (57.4%)
≥3	26 (42.6%)
Screw density	
<0.67	24 (39.3%)
≥0.67	37 (60.7%)
Fixation method	
Open	46 (75.4%)
Percutaneous	15 (24.6%)

SD, standard deviation; SINS, spinal instability neoplastic score; ECOG, Eastern Cooperative Oncology Group; LIV, lowest instrumented vertebra. * SINS 0–6 represents stable, 7–12 potential unstable, and 13–18 unstable. † number of instrumented vertebrae without metastases.

**Table 2:** Factors related to IRCs by log-rank test of Kaplan–Meier survivorship analysis.

Variable	No. of Patients	No. of Patients with IRCs	*p*-Value
Sex			0.733
Male	37	16 (43.2%)	
Female	24	11 (45.8%)	
Age (years)			0.279
<60	22	8 (35.4%)	
≥60	39	19 (48.7%)	
Body mass index (kg/m^2^)			0.777
<18.5	1	0 (0%)	
18.5–24.9	38	17 (44.7%)	
≥25	22	10 (45.4%)	
Primary tumor site			0.506
Lung	14	5 (35.7%)	
Kidney	12	6 (50.0%)	
Breast	8	2 (25.0%)	
Liver	6	2 (33.3%)	
Prostate	5	2 (40.0%)	
Thyroid	4	3 (75.0%)	
Colorectal	2	2 (100%)	
Miscellaneous	10	5 (50.0%)	
Number of involved vertebral bodies			0.992
1	49	22 (44.9%)	
2	7	3 (42.9%)	
≥3	5	2 (40.0%)	
SINS criteria †			0.325
Stable	2	0 (0%)	
Potential unstable	52	25 (48.1%)	
Unstable	7	2 (28.6%)	
Location of metastases			0.544
At or above T10	27	11 (40.7%)	
Below T10	34	16 (47.1%)	
History of radiotherapy			0.042 *
Yes	55	27 (49.1%)	
No	6	0 (0%)	
Preoperative ECOG scale			0.374
0–2	40	16 (40.0%)	
3–4	21	11 (52.4%)	
Postoperative ECOG scale			0.100
0–2	54	22 (40.7%)	
3–4	7	5 (71.4%)	
Location of the LIV			0.210
At or above T10	23	8 (34.8%)	
Below T10	38	19 (50.0%)	
Type of surgical procedure			0.073
Posterior fixation only	19	12 (63.2%)	
Posterior fixation with laminectomy	25	10 (40.0%)	
Posterior fixation with debulking procedure	17	5 (29.4%)	
Fixation length ‡			0.017 *
<3	35	21 (60.0%)	
≥3	26	6 (23.1%)	
Screw density			0.027 *
<0.67	24	14 (66.7%)	
≥0.67	37	13 (32.5%)	
Fixation method			0.074
Open	46	18 (39.1%)	
Percutaneous	15	9 (60.0%)	

IRC, instrumentation-related complication; SINS, spinal instability neoplastic score; ECOG, Eastern Cooperative Oncology Group; LIV, lowest instrumented vertebra. * Indicates statistical significance (*p*-value less than 0.05). † SINS 0–6 represents stable, 7–12 potential unstable, and 13–18 unstable. ‡ number of instrumented vertebrae without metastases.

**Table 3:** Incidence of IRCs with combination of fixation length† and location of the LIV.

	Location of the LIV		*p*-Value
	At or Above T10	Below T10	
Fixation length † <3	7/11 (63.6%)	14/24 (58.3%)	0.770
Fixation length † ≥3	1/12 (8.3%)	5/14 (35.7%)	0.005 *
*p*-value	0.007 *	0.105	

IRC, instrumentation-related complication; LIV, lowest instrumented vertebra. * Indicates statistical significance (*p*-value less than 0.0083) by Mann–Whitney U test. † number of instrumented vertebrae without metastases.

## Data Availability

The data underlying this article cannot be shared publicly because of the privacy of the individuals who participated in this study. The data can be shared by the corresponding author upon reasonable request.
